# Practical Treatise on the Art of Midwifery

**Published:** 1842-10

**Authors:** 


ART. III.

1. *Traité Pratique de l'Art des Accouchemens.* Par CHAILLY (Honoré), D.M. &c. 206 figures sur bois.—*Paris*, 1842. 8vo, pp. 784.
*Practical Treatise on the Art of Midwifery.* By Dr. CHAILLY. With 206 Woodcuts.—*Paris*, 1842.

2. *On the Theory and Practice of Midwifery.* By FLEETWOOD CHURCHILL, M.D. M.R.I.A. &c. &c. Illustrated by upwards of 100 highly-finished Wood Engravings by BAGG.—*London*, 1842. 8vo, pp. 479.

M. CHAILLY has been trained to practical knowledge in excellent schools and by equally good masters, and in the present volume he gives us the sum and substance of the doctrines and opinions of the well-known and highly-esteemed P. Dubois, whose pupil he has been for many years. He has added, too, the fruits of his own personal experience, derived from a long attendance at the Maternité and the Clinique d'Accouchemens of Paris; and to render his treatise complete, he has drawn occasionally upon the works of the most celebrated foreign writers, as Lachapelle, Desormeaux, Naegele, Velpeau, Stoltz, Moreau, &c. The object of the work is to give practical information; the various speculations and discussions upon merely scientific points that are entered into by various midwifery writers are purposely abstained from by M. Chailly, although their interest is not denied.

The first chapters of the work are dedicated, as they ought to be, to an account of the various organs engaged in the act of labour, and the diagnosis of normal pregnancy, from the commencement to the termination of utero-gestation. Upon the contested and still doubtful subject of the stethoscopic sign, termed "souffle placentaire," placentary murmur, M. Chailly observes that the cause of this sound has been attributed exclusively to the circulation of blood in the placenta, but that if this were the case we ought, in the same woman, always to hear the "souffle" at the same place during the whole period of pregnancy; it is not, however, he says, fixed at any particular point; besides which the same sound is heard when the uterus is developed by disease, and when the ovaries are enlarged, and in cases where there is no placenta, as after labour, when the placenta has been extracted. Bouillaud* designates this sound by the name of "souffle abdominal," and attributes it to compression of the large vessels, the aorta and iliacs. Haüs is of the same opinion.† Kennedy (on Obstetric Auscultation), and Rigby (System of Midwifery, p. 53), and the majority of modern observers have abandoned the opinion first promulgated by Kergaradec, that this so-called "souffle placentaire" is produced by the circulation of the blood in the spongy structure of the placenta. It is no longer doubtful, we believe, that this sound is not connected with the placenta, but that it depends upon the increased vascularity and peculiar arrangement of the uterine vessels during pregnancy, and hence the application of the term "souffle utérin" by P. Dubois and others.

We are gratified to find, from the remarks of M. Chailly (p. 184), that the induction of premature labour, in appropriate cases, has no

* Traité Clinique des Maladies du Cœur, pp. 274.

† Die Auscultation in Bezug auf Schwangerschaft, von Dr. C. J. Haüs.—Würzburg.

longer to contend with the mistaken prejudices that were formerly entertained against the practice by the French accoucheurs. The best among them now regard the practice, when properly and carefully limited, as a valuable and perfectly justifiable addition to our means of averting dangers that would be almost insuperable by other means. In addition to the testimony upon this very interesting subject, which the records of English writers would afford, we may observe that from the statistical account published by Stoltz in 1838, of 211 labours artificially induced, more than half the children were born alive, and M. Chailly says, scarcely 1 of the mothers died in 15. We are sorry it is not stated whether this mortality of the women was fairly to be attributed to the operation. H. F. Kilian's* cases, collected from various sources, afford less cause for apprehension as to the fate of the mother: of 161 cases in which premature labour was induced in different countries up to the end of 1831, 115 of the children were born alive, 46 died; 73 of the 115 continued to live and had good health; 42 died soon after birth, or their fate was unknown: 8 of the mothers died after the operation, but 5 of these from disease which had not the slightest connexion with the operation. In this country we have heard and read a good deal of the frequent success of the Cæsarean operation in France. Here is M. Chailly's evidence as to Paris: "We have not had within the walls of Paris a single example of a woman who has survived the Cæsarean operation." (p. 185.) In one case operated upon by P. Dubois, the woman died 17 days after the operation of tetanus, when everything promised a favorable issue. This is the longest period after the operation which any of the patients lived. Upon three or four occasions we have known artificial labour induced without any preparatory treatment. M. Chailly, and most continental writers, consider it very requisite. "The operation being decided upon, the woman should be prepared for several days before, by warm baths and emollient injections into the vagina." (p. 187.) Dubois performs the operation in the following manner: By means of a speculum he first brings the cervix uteri into view, and inserts into the os uteri a small and well-greased cone of prepared sponge, with a thread attached to it, to secure it externally. A piece of soft sponge is then introduced into the vagina large enough to fill it; the whole is supported by compresses and a T bandage. M. Dubois frequently, too, conjoins with these measures the exhibition of three doses of ergot. M. Chailly has almost always found uterine contraction take place in the course of an hour or two after the use of these means, and when the severity of pain indicates a sufficient dilatation of the os uteri, to permit the easy rupture of the membranes, the liquor amnii is evacuated by puncturing them with a pen, cut as if for writing, and the act of labour is induced. (p. 188.)

We cannot at all coincide with some of the opinions stated by M. Chailly upon the subject of abortion. In the first place he endeavours to show why it must be, and that it is the fact, that the hemorrhage that accompanies abortion is less abundant in proportion as pregnancy is far advanced. "Aussi l'hémorrhagie est-elle d'autant moins abondante, que la fausse couche a lieu plus près du terme." (p. 203.) Again, as to the prognosis of abortion with hemorrhage, "as to the mother it is the more

* Die Operative Geburtshülfe. Von Dr. H. F. Kilian.—Bonn, 1834. Erster Band. pp. 298.

serious (plus grave) the less the pregnancy has advanced." (p. 205.) Now that the very reverse of these two assertions is practically true in the great majority of cases, we have no doubt whatever, and we confess we thought there was upon this point at least a unity of opinion among practitioners. In the treatment of the first period of threatened abortion, and for the purpose of preventing it, great confidence is attached by M. Chailly to the use of repeated enemata, with from 15 to 20 drops of laudanum administered every half hour till the symptoms have subsided. In many cases he has known very severe hemorrhage occur in the early months, and still pregnancy has gone on to the full period. This is in accordance with our own experience, and we advert to the sentiment of M. Chailly upon this point, for the purpose of diminishing the belief that is entertained by many that abortion must of necessity follow if the hemorrhage is at all abundant. This opinion is mischievous, because when it is held, the practitioner thinks little or nothing of attempting that in which he may still succeed, the prevention of miscarriage. The care that M. Chailly takes to limit the use of cold during hemorrhage is very prudent, for in ordinary practice no remedy is more frequently abused. He also very properly inculcates the propriety of keeping the upper parts of the body warm by every possible means, in severe cases of hemorrhage, while cold is being applied to the lower part of the abdomen and the thighs. More than one case has fallen under our notice in which if this rational principle had been acted upon, the flickering spark of life that was still remaining might perhaps have been preserved and health restored, when the little of power that was left was exhausted by the too long continuance of cold. In severe cases of hemorrhage, M. Chailly advocates the practice of plugging the vagina, an expedient which our readers are doubtless aware is not approved of by all English authorities, from the apprehension of converting an external into an internal hemorrhage. We are not prepared to deny that this objection to the plug has some foundation, but infinitely too little, we affirm, to deter us from the use of it, at any period of pregnancy. He objects, too, to the practice of rupturing the membranes in cases of severe hemorrhage in the early months of pregnancy, and points out very properly the importance of the ovum being expelled unbroken. At a later period of pregnancy we may be justified in rupturing the membranes, because if then the placenta is not expelled, the size of the uterus will permit the introduction of the hand for the purpose of removing it. In this opinion we entirely agree; but we could quote some opponents to it among very respectable English professors. In commenting upon the physiological phenomena of labour, M. Chailly observes that in obstetrical language the words pain and contraction are employed as synonymes, because in most women pain is inseparable from uterine contraction. Some women, however, are delivered with very little pain, and still efficient contraction of the uterus must have taken place. This is true, but the fact is rare. In one case that recently occurred to us of a shoulder presentation, in which we found very great difficulty in introducing the hand, on account of very rigid uterine contraction, the patient suffered scarcely any pains.

*Mechanism of spontaneous delivery.* Upon this subject M. Chailly states, in opposition to some authorities, that the mechanism of spontaneous delivery is exactly the same in all positions of the summit of the head, whether the occiput looks to any point of the right or left lateral half of the pelvis. There is, however, a slight difference in the anterior and posterior positions. In the latter the movement of rotation, which brings the occiput under the arch of the pubis, is more extended, and besides, these positions may sometimes, but very rarely and as exceptions, be converted into permanent posterior positions. The general law, however, remains valid, that spontaneous delivery is effected in obedience to the same rule, whatever may be the part of the brim of the pelvis with which the occiput may be in relation. We cannot sufficiently admire the simple, just, and practical manner in which the whole subject of the mechanism of labour is treated. M. Chailly never allows himself to be drawn from his subject by mathematical explanations and illustrations which some of his and of our brethren, too, are so fond of indulging in; they can never be necessary, and must always be "caviare to the multitude" of practical enquirers. The most experienced may sometimes, says M. Chailly, be deceived in the following manner: We have seen several such cases. Without any other phenomena of labour there may be regular uterine pains. Upon examination per vaginam we may feel assured that the process of labour is commenced. The cervix uteri is open, the membranes form a tense tumour, the pains go on for some hours, and then a calm succeeds, and true labour does not occur for several days. M. Chailly has frequently witnessed these premature contractions, and occasionally several times in the same patient during the last fortnight of pregnancy. "How can we distinguish at first this commencement of labour from a regular labour that is to go on to the expulsion of the child? I do not believe it is possible to guard against this error; time alone can enlighten the accoucheur upon this point." (p. 291.) This is true, and it teaches us not to risk the confidence of the patient by hasty and decisive opinions as to the termination of a labour. As Horne Tooke advised in politics, so ought we in midwifery practice, "to deal in generals," and learn the art of equivocating by consolotary phrases which tie us to no fixed time. M. Chailly agrees with Kilian* that, although rupture of the perineum is by most practitioners but little feared after the exit of the head, and therefore no support is given to it during the passage of the shoulders, most lacerations of this part are caused when the shoulders are expelled. The experience of thirty very active years in midwifery practice leads us to a different conclusion. When the perineum is ruptured, we believe, the accident is very generally, we scarcely refrain from saying always, caused by the passage of the *head* of the fœtus. The following hint as to the performance of a comparatively trifling duty is not without value or necessity. When the funis contains a larger quantity of the gelatinous matter than usual, it is useful, before we apply a ligature, to press out the lymph it contains with the fingers, or to give an exit to it by small punctures. Without this precaution, after the spontaneous escape of the fluid, the ligature fails to act upon the divided vessels, and the blood escapes from the umbilical arteries, and endangers the life of the child. M. Chailly is surely wrong, or we and all other observers are, in asserting that "there is in twin cases almost always vascular communications between the two placentas." (p. 307.) Such is very rarely the fact. The following remark and caution may appear unnecessary and not very complimentary to the profession. We can corroborate M. Chailly's statement, however, that it

* Loc. cit. "Von dem Schutze des Dammes." Erster Band. p. 171.

has not unfrequently happened that practitioners have felt confident of the existence of, and have treated cases for, puerperal inflammation, by bleeding, baths, &c. &c., when the only mischief that existed was a bladder over-distended with urine, which had altogether escaped attention from gross carelessness. He believes, contrary to the general opinion, that the ergot has the power of causing uterine contraction when it has not before been manifested. "This medicine, however it may be administered, is frequently rejected by vomiting. It then should be given in a clyster, and it is thus even more rapidly absorbed, than when given by the stomach, and acts more directly upon the uterus." (p. 324.) This is a valuable bit of information, *if* it be correct. We should have liked a little detailed proof of this alleged fact. In irregular, ineffectual, and exhausting uterine contractions much confidence is placed in repeated enemata of injections of a quarter of a pint of warm water, and 15 drops of laudanum, gradually increasing the quantity to 60 or 80 drops, and thus repeating the injections every quarter of an hour if necessary, but "in general, a few minutes after the first injection, the pains diminish, a calm succeeds, refreshing sleep, and a return of more regular and effective pains occurs." (p. 329.)

*Anterior obliquity of the uterus.* By placing the patient in a proper position, and by the application of a bandage, any inconvenience arising during labour from this not unusual malposition of the uterus is usually overcome. "But sometimes it is necessary to draw the cervix uteri forwards by the fingers of one hand, while the uterus is raised with the other. This reduction of the cervix should be effected during the absence of pain, but the fingers should keep the cervix forwards during contraction." (p. 335.) We are inclined to speak even more guardedly upon this subject than our author does. In the very great majority of cases of anterior obliquity, Dr. Gooch's "tincture of time" is the best and the efficient remedy. It is true we may often draw the cervix forwards during the absence of pain, when it is very much tilted backwards from the anterior bearing of the uterus itself, but until nature herself has, perhaps by long efforts, placed the parts in a more favorable position, we can very seldom keep the cervix forwards with the fingers without such a degree of force that the nature of the case neither justifies nor requires. M. Chailly attended the labours of two paraplegic women at the Clinique. Neither of these patients required any particular attention. "Still this disease may impede the regular uterine action and render assistance necessary. It might be the same with hemiplegia." There is a paucity of information upon this subject in midwifery records. A case recently occurred at a London hospital of a paraplegic woman, who went through the act of labour without assistance or difficulty. At page 359, Dr. Montgomery's opinion upon the subject of spontaneous amputation of the limbs of the fœtus in utero is misstated. Dr. Montgomery (vide Signs and Symptoms of Pregnancy, p. 321, et seq.) does not attribute this event to "constriction of the funis" alone. He admits it as an occasional cause of this effect; but he proves by his own experience and that of others, that the most frequent cause of this injury to the limb of the fœtus arises from the formation of firm bands or threads of organized lymph, which act as a ligature. We have now before us a preparation of a five months' fœtus. The funis is coiled twice tightly round the neck and once round the left arm, which it has deeply indented. The death of this fœtus probably arose from the circulation in the cord being obstructed. Had it lived to the full period the arm would no doubt have been partially if not entirely amputated from the firm pressure inflicted upon it.*

M. Chailly considers it "perfectly useless" to establish a diagnosis between those cases of uterine hemorrhage which depend upon attachment of the placenta over the os uteri, and those which are what we term "accidental," inasmuch as the only indication for our practice is the degree of severity of the hemorrhage from whatever cause it may proceed. In this country we think differently, and, with respect be it said, we believe more justly upon this point. And though at page 368 this diagnosis is said to be "complètement inutile," at page 376 sufficient reasons are given to prove the contrary. The plug is mentioned as "un moyen précieux" in severe hemorrhage during the latter months. In cases of rigidity of the os uteri the application of extract of belladonna is highly spoken of. We know but little of this practice in England. "I have frequently seen the application of belladonna successful. We should use the extract of the consistence of soft wax. A small portion of it should be placed upon the nail of the index-finger, and carried within the cervix uteri. The heat and mucous moisture dissolve it, and it may be spread easily over the internal surface of the os uteri. When this remedy is successful it generally acts very quickly. The cervix softens and dilates in ten or fifteen minutes." (p. 396.) The praise bestowed upon this practice is tempered by candour, for it is confessed that it often fails. Notwithstanding the high authorities opposed to us, we feel quite con vinced that M. Chailly's disposition to leave what are termed malpositions of the head to nature, rather than have recourse to manual interference for the purpose of placing the head in the strictly natural position, is very wise and very prudent. We are gratified to find at p. 424 that M. Chailly deprecates the practice of many of his brethren, who from very weak scruples, refuse to open the head of a living child, whatever may be the danger to which the mother is exposed by the continuance of the labour. The doctrine of English accoucheurs, upon this very delicate and responsible point, are liberally preferred to those which generally prevail in France and other parts of the continent. M. Chailly has frequently seen the "cephalotribe" of M. Baudelocque employed with the greatest success in cases where formerly the Cæsarian operation would have been deemed necessary. No doubt there must be difficulty and danger in the employment of this instrument, but we think it deserves more attention than it has met with here. We object to the following rule that is laid down for the application of the forceps. "The hand opposed to that which holds the handle of the instrument should always be introduced, excepting the thumb, into the maternal passage, to direct the blade and protect the vagina against the contact of the instrument." (p. 446.) This would always be very painful to the patient, generally difficult for the practitioner, and can rarely if ever be necessary. Two fingers will sufficiently and safely guide the blade of the forceps. After the example of M. Dubois, M. Chailly prefers introducing the blades of the forceps first at the back of the pelvis, and then brings them round to the point they are to be definitively placed in by moving the handle of the instru-

* In our Ninth Volume, p. 564, we have recorded a similar case from an American Journal.

ment. M. Dubois, too, and M. Chailly have strong objections against showing the forceps to the patient before they are employed. We, on the contrary, think and have always taught our pupils that it is very prudent to do so. The patient knows that instruments are to be employed. She thinks that all instruments are cutting instruments. Show her the forceps; tell her that they are, what they really are, a long pair of hands, and her fears are calmed. We at least have always found this to be the case. The following passage evinces a nicety of ear and a degree of stethoscopic tact which we quite envy:

"The accoucheur may be called upon to apply the forceps, when the head, long engaged in the pelvis, may be the seat of a considerable sero-sanguineous tumour which marks the character of the position. It is in such a case that auscultation renders us such great service. In fact, if the 'summum' of intensity of the pulsations of the fœtal heart is heard directly forward, the case is one in which the occiput is either to the pubis or the sacrum, and the application of the forceps is the same. If the 'summum' of intensity of the pulsations is to the left, there is every reason to believe that the position is with the occiput anteriorly to the left ilium," &c. &c. (p. 478.)

M. Chailly proceeds much further with these stethoscopic distinctions than we deem it necessary to quote. That no man can establish them we will not venture to assert; but we do most positively that, for the purposes of general practice, such rules, as guides for the manner of applying the forceps, are utterly worthless and unavailable. The objections that are urged against the practice of separating the membranes from the uterus when the hand is introduced for the purpose of turning the fœtus, appear to us practically sound. It is better, easier for the practitioner, and safer for the mother and child, to "rupture the membranes at the centre when the hand enters the uterus." (p. 511.) Some writers lay down very exact rules as to the manner of grasping the fœtal limbs in the operation of turning, and the precise position of the operator's fingers, as well as the parts of the fœtus on which they should be placed. All this M. Chailly very properly smiles at. "We grasp the limbs how we can. (p. 515.) In general we can turn as easily by grasping one extremity as with both." Upon the subject of the Cæsarean operation M. Chailly's sentiments are exactly in keeping with the invariable rule of British midwifery practitioners, and strongly opposed to many among the French and Germans even of high repute. "I will never perform the Cæsarean operation, except in cases of pelvic contraction, that render the mutilation of the fœtus impossible." (p. 553.) Every well-informed accoucheur is aware how much we are indebted to Madame Lachapelle (Pratique des Accouchemens), for clear notions respecting the passage of the head through the pelvis in face presentations, as well as for proving that the efforts of nature are usually sufficient. M. Chailly agrees with Madame Lachapelle. When the breech or feet present, and the back and occiput of the fœtus are directed towards the back of the mother, "the delivery in this posterior occipital position is not so difficult as is generally imagined; and when much difficulty is experienced in turning the back of the fœtus towards the mother, we should not obstinately persist in effecting this change." (p. 613.) Violence can never be justifiable to alter this unfavorable position of the fœtus, but very generally the attendant may and ought to prevent it if he has the whole management of the case.

We have noted in M. Chailly's book many other passages from which we might select good practical remarks; but we must stop. We must, however, in common justice, declare our opinion that the work merits much more than the cold praise of being respectable. It would be attractive if it were merely from the fact of its giving to the profession the practical opinions of M. Dubois. It is additionally so from the information afforded by the author himself, and the clear style in which it is conveyed.

The object of Dr. Churchill's volume is to offer to the student in midwifery a work, embracing the modern discoveries in the physiology of the uterine system, with all the recent improvements in practice, in a condensed form, amply illustrated, and at a moderate price. It contains ample evidence of the industry with which the author has consulted every authority of eminence to bring his work up to the present state of science, and he confesses, generally, in the preface that he has not hesitated to avail himself of the labours of Drs. Ramsbotham, Rigby, and others. It would have been well, we think, if more precise acknowledgment had been granted to Dr. Ramsbotham, for we observe that many of the woodcuts are copied exactly from his work: others are merely reversed. Dr. Churchill's work is upon the whole a fair and instructive compilation; but, as Malthus says, "it is born into a world already possessed," and we cannot conscientiously say that it will bear comparison as a practical guide, with other works that have recently appeared upon the same subject, and which we have noticed.

---

